# Examining the Importance of the Teachers' Emotional Support for Students' Social Inclusion Using the One-with-Many Design

**DOI:** 10.3389/fpsyg.2016.01014

**Published:** 2016-06-30

**Authors:** Zarina Hogekamp, Johanna K. Blomster, Aslı Bursalıoğlu, Mihaela C. Călin, Melis Çetinçelik, Lauge Haastrup, Yvonne H. M. van den Berg

**Affiliations:** ^1^Department of Basic Psychological Research and Research Methods, University of ViennaVienna, Austria; ^2^Department of Psychology, University of OsloOslo, Norway; ^3^Department of Psychology, Koç UniversityIstanbul, Turkey; ^4^Institute of Health and Society, University of WorcesterWorcester, UK; ^5^Department of Psychology, University of Southern DenmarkOdense, Denmark; ^6^Behavioural Science Institute, Radboud UniversityNijmegen, Netherlands

**Keywords:** dyadic analysis, one-with-many design, teacher emotional support, social inclusion, academic functioning

## Abstract

The importance of high quality teacher–student relationships for students' well-being has been long documented. Nonetheless, most studies focus either on teachers' perceptions of provided support or on students' perceptions of support. The degree to which teachers and students agree is often neither measured nor taken into account. In the current study, we will therefore use a dyadic analysis strategy called the one-with-many design. This design takes into account the nestedness of the data and looks at the importance of reciprocity when examining the influence of teacher support for students' academic and social functioning. Two samples of teachers and their students from Grade 4 (age 9–10 years) have been recruited in primary schools, located in Turkey and Romania. By using the one-with-many design we can first measure to what degree teachers' perceptions of support are in line with students' experiences. Second, this level of consensus is taken into account when examining the influence of teacher support for students' social well-being and academic functioning.

## Introduction

Students spend on average 7751 h with their teachers during their primary and lower secondary education (Organisation for Economic Co-operation Development, 2013). Through the many hours of instruction and interaction, teachers help students acquire academic knowledge and skills. However, teachers also prepare children for later functioning in society by teaching students to successfully navigate in the social world, both in and outside of the school. Previous studies already showed that teacher's emotional support is very important for students' social functioning and academic engagement (Farmer et al., 2011). Unfortunately, these studies did not look at the constant interplay between teachers' intended level of support and a student's experienced support. Therefore, there is much important information left unstudied. Arguably, teacher support may only be of importance for student's well-being when a teacher's intention to be supportive is also experienced as supportive by the student. In the current study, we will therefore use a dyadic analysis strategy called the one-with-many design to gain a better and more detailed insight in the importance of teacher support for students' academic and social functioning.

### Students' social and academic adjustment at school

Students not only interact with their teachers at school, but also interact to a large extent with their peers. Therefore, school is not only a place where children learn to read and write, it is also one of the most important contexts in which they acquire social skills (Hughes, 2012). The classroom is where children interact the most with their peers, and through these interactions children develop social competence (Hughes, 2012). Furthermore, the school is a place where children experience feelings of social inclusion for one of the first times. However, the classroom is often also the context where some children experience being socially excluded for the first time. The consequences of being socially excluded are severe both for the individual and for the society as a whole. Excluded people show reduced abilities to self-regulate, which leads to aggression or even crime (Baumeister et al., 2005; United Nations Educational Scientific Cultural Organization, 2010).

Feelings of social inclusion or exclusion are not only important for children's general well-being and social-emotional development. Importantly, feelings of social inclusion also make students benefit more from education (Holz, 2004). For instance, previous research has found that academic engagement of students correlated with feelings of relatedness with teachers and parents (Skinner et al., 2009), and students' school engagement has been found to be an important predictor of their school dropout and academic success in their later education (Croninger and Lee, 2001; Fredricks et al., 2004; Balfanz et al., 2007; Hafen et al., 2012). Importantly, research has shown that social exclusion is likely to promote gradual disengagement as students progress from primary or elementary level to middle school and high school (Skinner et al., 2008; Martin, 2009). Thus, it is very important for children's general well-being and academic success to feel safe and socially included at school.

### Affective quality of teacher–student relationship

Numerous studies have shown that the affective quality of teacher–student relationships is predictive of students' academic functioning and performance (for a review, see Hamre and Pianta, 2006). In addition, students who experience high levels of positive and supportive interactions with their teachers are better liked and more accepted by their peers (Hughes and Kwok, 2006; Hughes, 2012). Providing emotional support is one factor through which teachers can impact students well-being (Buyse et al., 2009), and academic engagement (Skinner et al., 2009). Emotional supportive teacher–student relationships involve teachers being emotionally positive toward students, and setting clear social rules while still allowing students to develop their own social norms (Farmer et al., 2011; Hughes, 2012).

Previous studies have mainly looked at one-sided perceptions of teacher–student relationships, namely teachers' own perceptions on the level of support they provide to students. Student experiences of teachers' emotional support is often not examined, nor have studies looked at the correspondence between students' experienced support from their teachers and teachers' intended level of support. This means that a great amount of information is left unexplored: whilst teachers might aim to provide emotional support, whether this support is in fact perceived and thus experienced by students is of utmost importance. Perceptions of emotional support can validate its receival and can ensure pupils benefit from the aforementioned positive outcomes. In a study examining the perceived therapeutic alliance by both therapists and their clients, Marcus et al. (2009) found that therapists' with general tendency to form strong therapeutic alliance—as reported by their clients—had clients with better outcomes. However, therapists' own perception of their alliances were not associated with better therapeutic outcomes. In this particular case the clients' opinions of their therapist were associated with better outcomes, whereas his own opinion was not. This information can be used to inform therapists of their work-efficacy and inform interventions to enhance clients' view of their therapeutic alliance with their therapists. These results underline the importance of studying reciprocity in order to detect elements that influence outcomes for any side of a dyad.

Therefore, the first step is to explore the level of correspondence and reciprocity between teacher's own perceptions of emotional support and students' experienced support from their teacher. Next, we will examine whether students' academic and social functioning can be explained by teacher's intended level of support, students' experienced support, or by the reciprocity in teachers' intended and students' experienced level of support.

With this in mind, the present study will answer the following questions:

1. Does emotional support toward the student as perceived by the teacher correspond with students' experienced emotional support?
  - On a general level, are teachers who report to provide high degrees of emotional support also perceived as giving high levels of such support?
  - On an individual level, if a student experiences a lot of emotional support from their teacher, relative to the level of his/hers classmates, does the teacher then also report to give more emotional support to said student, relative to other students?
2. How is teacher's emotional support as perceived by both teachers and students, associated with students' social inclusion and academic functioning?
  - To what degree is teachers' reported support toward his students in general associated with students' social inclusion and academic functioning?
  - To what degree is teachers' reported support toward an individual student associated with the social inclusion and academic functioning of that specific student?

## Methods

### Participants

We aim to recruit a sample of 15 teachers and their students (15 teachers × 25 students = 375) from Grade 4 (age 9–10 years) in public primary schools from each country selected for inclusion—Turkey and Romania.

Education systems in Turkey and Romania are similar in their structure and develop in a predominantly collectivist cultural background (Hofstede et al., 2010). In both countries, primary education is mandatory and free of charge for all citizens. The cycle develops over 4 years and the starting age is 6 years old in both cases. The pupil-teacher ratio is estimated at 18 pupils per teacher in Romania as opposed to 20 pupils per teacher in Turkey (UNESCO Institute for Statistics, 2016). In practice however, the National Law for Education (2011) in Romania assumes that classroom size varies across teachers but it is not meant to exceed 28 students and 25 is considered optimal. In Turkey, according to the data from Ministry of National Education (2016), the average number of students per teacher in the educational year 2015–2016 is 18. However, the hitherto recruited classrooms had an average size of 23. Having a mean of students per teacher larger than that of the country indicates the reliability of this number.

The ongoing data collection yields smaller classes in Romania as opposed to Turkey. We therefore aim to collect the estimated student sample across all teachers. Under determination of alpha at 0.05, power of 0.7 and medium cohen's f^2^ effect size of 0.15 a standard multiple regression sample size calculation in G^*^Power yielded 33 for the teacher sample size. In additional support of our sample aim we refer to a previous study (Marcus et al., 2011) which used a one-with-many design with a sample of 14 therapists and 398 substance use adolescents, making our teacher sample large enough to provide sufficient power. Limitations of applying standard power analysis on a one-with-many design are discussed below to further elaborate on our teacher and student sample size aim.

### Measures

#### Teacher–student relationship scale (TSR)

The TSR (Gehlbach et al., Unpublished manuscript) includes teachers' and students' perspective of their relationship (see Table 1). Teachers and students items are correspondent hence suitable for a one-with-many design to assess both parties perceptions of their relationship. The scale measures both negative (five items) and positive (nine items) aspects of the relationship (Gehlbach et al., Unpublished manuscript). The positivity and negativity items are treated as two different subscales and as such will have their own score calculated, i.e., mean scores will be given for each teacher and each of his students. Examples of matching student and teacher items are “How motivating are the activities that < teacher's name> plans for class?,” and “How motivating does < student's name> find the activities that you plan for class?” Gehlbach et al. (Unpublished manuscript) report on means and standard deviations for each of the subscales at two different time points. The provided standard deviations for the four subscales ranged from 0.52 to 1.01.

**Table 1:** **Psychometric properties of the scales used**.

**Name of Scale**	**Measures**	**Number of items**	**Reported by**	**Scoring system**	**Cronbach's α**
Teacher–student relationship scale	Teachers' emotional support	14	Teacher-report and student self-report	5-point Likert scale	0.90 (teacher perspective on positivity)
					0.78 (teacher perspective on negativity)
					0.92 (student perspective on positivity)
					0.78 (student perspective on negativity)
Social inclusion assessment instrument	Social inclusion	26	Student self-report	5-point Likert scale	0.87
Classroom peer context questionnaire	Classroom relations in the form of comfort, cooperation, conflict, mutual affection, and cohesion	31	Student self-report	5-point Likert scale	0.87 (comfort)
					0.82 (cooperation)
					0.87 (conflict)
					0.76 (cohesion)
					0.82 (isolation)
Peer nomination measure	Students relationship	10	Peer-nomination	Nomination by each student	N/A
Engagement vs. Disaffection with learning	Student engagement in the classroom	10	Student self-report	4-point Likert scale	0.72 (behavioral engagement)
					0.82 (emotional engagement)

#### Social inclusion

Students' social inclusion will be assessed using two measures and one peer nomination method.

##### Social inclusion assessment instrument (SIAI)

The SIAI (Rinta et al., 2011) is a self-report, 26 item scale that measures social inclusion among students in the classroom. It uses a 5-point Likert-type scale with smiley faces, ranging from a sad face (“I don't agree”) to a happy face (“I agree”), with a neutral face in the middle (Rinta et al., 2011). This kind of response scale has been shown to work well in cross-cultural contexts (Islam and Rashid, 2012) and among special needs and migrant children as well (Rinta et al., 2011). Means and standard deviations will give scores for social inclusion in each classroom.

##### Classroom peer context questionnaire (CPCQ)

The CPCQ (Boor-Klip et al., 2016) is a 5-point Likert scale measuring classroom climate with a total of 20 items. The five underlying factors are: comfort, cooperation, conflict, cohesion and isolation. An example item from the comfort factor is “In this class, I feel comfortable.” Means and standard deviations will be computed as a score for classroom climate.

All items in the questionnaire are either directed toward all classmates (class orientation) or individuals (personal orientation), which assesses student's peer-contexts (Boor-Klip et al., 2016).

##### Peer nomination measure

Peer nominations measure classroom social relations (Cillessen and Marks, 2011). This will be assessed using 10 items measuring social inclusion and behavior. The questions and the different subscales can be seen in Table 2. These nominations have been chosen because they represent social positions relevant for the concept of inclusion.

**Table 2:** **Peer nomination items and subscale**.

**Items**	**Subscale**
Who do you like best?	Acceptance
Who do you like least?	Rejection
Who are the most popular children in this class?	Popularity
Who are the least popular children in this class?	Popularity
Who are your best friends?	Friendship
Who would you say helps others a lot?	Cooperation
Who do you think cooperates well with others?	Cooperation
Who bullies others?	Bullying
Who is being bullied?	Victimization
Who would you say gets in fights often?	Aggression

The children will be presented with a peer nomination question (see Table 2), followed by nine numbered lines on which they can write the coded names of the peers they wish to nominate for that category. They will be given a list of codes for each peer additionally to this peer nomination measure. Children are allowed to nominate as many or as few of their classmates as they want, but not themselves or children outside of their classroom. The number of nominations each child receives per item will be summed up and standardized within classrooms, i.e., subsequently z-scores will be computed as a score for overall classroom social relations. Respectively z-scores less than −3 and bigger than +3 will be truncated (Tabachnick and Fidell, 2007).

#### Academic functioning

Academic functioning will be assessed using two different measures. A student-report measure for academic engagement and a teacher report for academic performance.

##### Engagement vs. disaffection with learning

The engagement vs. disaffection with learning scale measures students engagement in the classroom and has scales for student and teacher perspective. The scale operationalizes engagement in learning into four distinct components: emotional engagement, behavioral engagement, emotional disaffection and behavioral disaffection (Skinner et al., 2009). For this study the emotional and behavioral engagement subscales will be used, gathered from the student perspective. This gives a total of ten items, scored on a four point Likert-type scale. The complete scores for students will be reported as means and standard deviation in each classroom.

##### Academic performance items

Two items were included in the teacher survey in order to measure students' academic performance. These two items were “Compared to the other students, how well does this student do in language?” and “Compared to the other students, how well does this student do in maths?”

### Procedure

Recruitment and data collection have commenced in April and developed over the months of May and June 2016. Data has been collected in classrooms, using paper questionnaires for students and online as well as paper questionnaires for teachers. All measures are in English and have been translated and back translate to both Turkish and Romanian.

Ethical approval has been obtained from the Ethics Committee Social Sciences (ECSS) from Radboud University in late January and it has been followed by approval from each university affiliated with the junior researchers collecting data. Additionally, the project was granted ethical approval in Turkey from Koç University and from the concerning department of the Ministry of Education for İstanbul. The application was made in December and the approval was obtained in late February. Consent forms were sent to schools in March, and data collection initiated in April. In Romania, the Regional Educational Division of the Ministry (RO: Inspectoratul Judeţean Argeş—ISJ) in county of Argeş, Romania granted approval to conduct the study. Primary schools located in Piteşti (capital city of Argeş) were contacted right after. Consent forms were sent early in April and participants were given 3 weeks to return the completed forms. Data collection commenced on the 9th of May.

Overall, in both countries, data collection has been done in a 3-month process which has started in April and is scheduled to end in June. The time of data collection coincided with the end of the school year which was a change from our initial aim of aggregating data at the beginning of Semester 2. The difference in the times of the year could have had an effect on students' level of enthusiasm toward school, hence, affect their need to communicate with their teacher. The students who might have completed the questionnaire in the middle of the school year might have felt more dedicated to their class and have a more responsive relationship with their teachers than the students who completed it toward the end of the school year.

The recruitment process consisted in sending letters to selected schools, informing on the study's design, methods, procedure and information on privacy and confidentiality matters. Follow-up calls to teachers were made shortly after and active consent was sought from parents.

Data collection was scheduled for an hour for each classroom. The researcher distributed the questionnaires to all participating pupils and gave an introduction along with verbal instructions and reassurance of anonymity and the right to withdraw. A story about a secret mission of famous cartoon characters minions was introduced and participants were then encouraged to complete their questionnaire in silence. Teachers were also given their paper questionnaires about each participating child and were encouraged to complete them at the same time as children did.

## Proposed analyses

### One-with-many design

In a reciprocal one-with-many design both the teacher and the student report on an outcome (e.g., emotional support). Variances can be estimated for both perspectives separately. Specifically four variances, two at the teacher level and two at the student level, will be estimated.

For teachers we will calculate the teachers' perceiver variance. This estimate indicates the degree to which a teacher reports to provide equal levels of emotional support across all of his students, thus the *assimilation* in his rating of provided emotional support across his students. Additionally the teachers' partner variance is obtained by calculating the means of ratings of each student. This indicates consistency in students' ratings of their teacher and thus their *consensus* as a group. Both measures will be utilized to give insight into the teacher–student relationship on a generalized, i.e., classroom level.

The students' variance estimates will give insight into the teacher–student relationship on a dyadic, i.e., individual level. The teacher relationship variance indicates *uniqueness*, i.e., the degree to which a teacher reports to provide an especially strong emotional support toward an individual student. The student relationship variance indicates *uniqueness*, i.e., the degree to which a student reports to obtain an especially strong emotional support from his teacher.

The single wave data can be estimated with multilevel modeling. The multilevel modeling framework takes into account the nestedness and non-independence of the data and will be used to estimate the different variance components introduced above (Kenny et al., 2006). Specifically a two level model, with teachers on the upper and students on the lower level, will be utilized. In the current demonstration version 21 of SPSS will be used to analyse the data.

#### Variance partitioning

In the reciprocal one-with-many design both teachers and students provide scores for emotional support by completing the TSR questionnaire. To later be able to indicate which of them provided a score the two intercept approach (Raudenbush et al., 1995) is used. To do this two dummy variables will be created to denote the provider of a score. Hence we create one dummy variable, T, which is coded 1 if the data are provided by the teacher and 0 if the data came from the student. Respectively a student dummy variable, S, will be created which is coded 0 if the data are provided by the teacher and 1 if the data is provided by the student. This way one intercept will be specific to teachers' ratings and one for students' ratings (Marcus et al., 2009).

Due to reciprocity and hence the introduced dummy variables variance partitioning is executed using a specific data structure. Specifically as each lower student level unit is embedded in a dyad and as each dyad includes two scores for emotional support there will be two rows per dyad indicating reciprocal emotional support scores including two columns for the newly created dummy variables, which indicate who provided the scores. The SPSS syntax to attain variance partitioning (Marcus et al., 2009) is provided in the **Appendix**.

### Reciprocity

**Figure 1:**
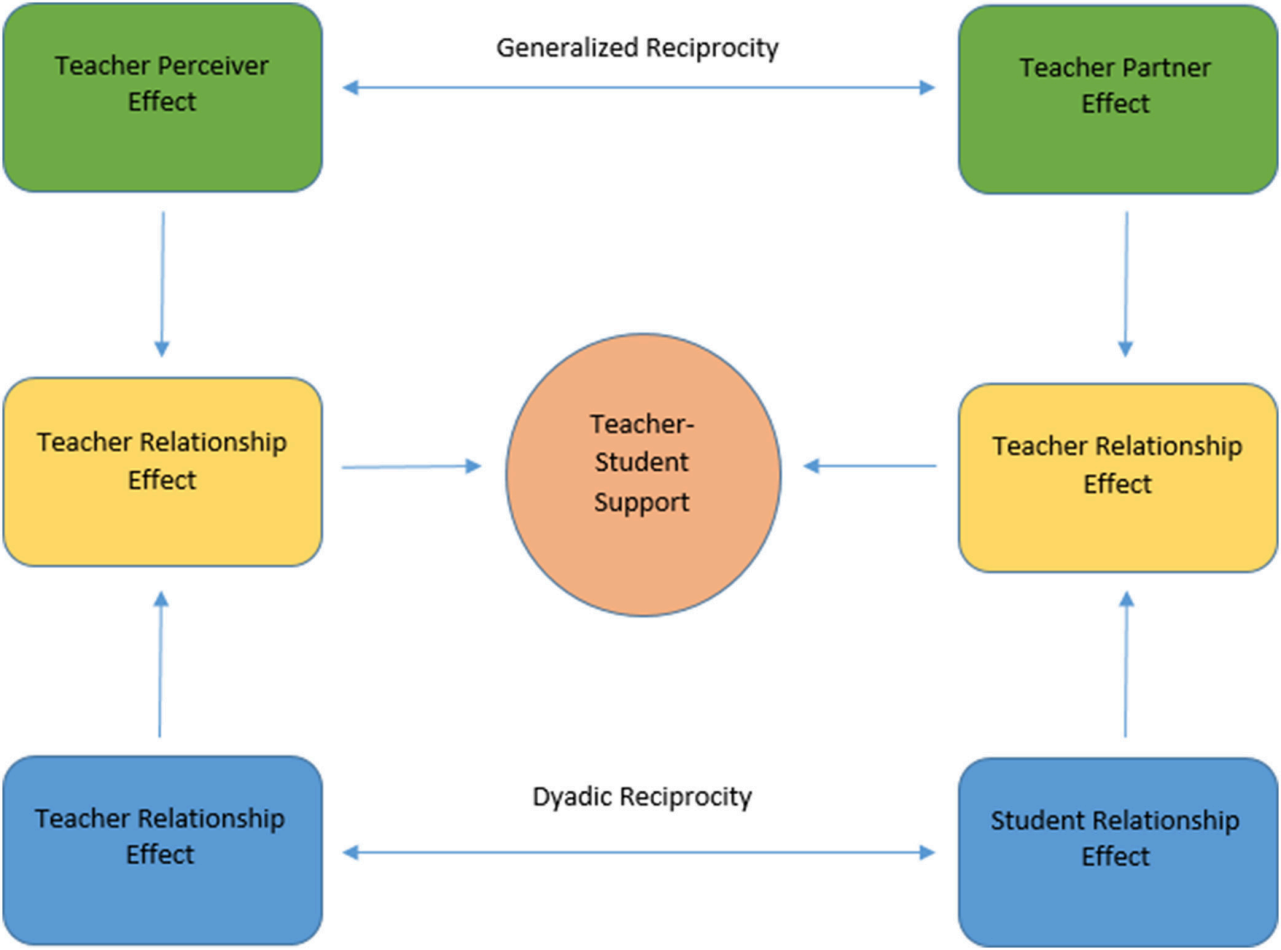
**Variance components of the teacher–student relationship derived from a reciprocal one-with-many-design**. Adapted from Marcus et al. (2009).

Research question 1a and 1b address correspondence of teachers' and students' perceptions of emotional support. In a second step, using the variance estimations, we will examine the correspondence between teachers' and students' report on emotional support at a generalized and dyadic level (see Table 3 and Figure 1). We do this by correlating the variance components that we attained by the variance partitioning step. We first estimate the generalized reciprocity by correlating the teachers' partner variance (reflected in student reports) with the teacher perceiver variance (reflected in teachers' reports). This suggests whether teachers who report to provide strong emotional support are backed-up in their view by their students. Next, we will examine the dyadic reciprocity by correlating the two relationship variances (reflected in both teachers' as well as students' reports). This suggests whether a teacher who reports to provide a uniquely strong emotional support to a particular student is in turn seen as emotionally supportive by that particular student.

**Table 3:** **Estimated effects by a one-with-many analysis of teacher emotional support**.

**Variance partitioning**	**Variance Source**	**Question answered by variance**
**TEACHER's EMOTIONAL SUPPORT**		
Teacher partner variance	Student report	Consensus: Do students report similar emotional support toward their teachers?
Student relationship variance	Student report	Uniqueness: Do students report unique emotional support provided by their teachers?
Teacher perceiver variance	Teacher report	Assimilation: Do teachers report to provide similar emotional support across their students?
Teacher relationship variance	Teacher report	Uniqueness: Do teachers report unique emotional support toward their students?
**RECIPROCITY CORRELATIONS**		
Generalized reciprocity	Teacher perceiver correlated with teacher partner	Are teachers who report strong support perceived to provide strong support by their students?
Dyadic reciprocity	Student relationship correlated with teacher relationship	If a student reports an especially strong support toward a teacher, does the teacher also report an especially strong support toward the student?

### Emotional support and student outcomes

Research question 2a and 2b address the influence of teachers' emotional support on students' social inclusion and academic functioning. In this last step of the analyses we will examine associations between the emotional support ratings given by teachers and students and four measures of outcome: social inclusion, classroom climate, hierarchy in classrooms and students' academic engagement. To analyse these student outcomes we will use linear multiple regression analyses.

The average of the outcomes across all students within each teacher will be predicted using the teacher variance components (e.g., teacher perceiver and partner effects; see Table 3). This way we can answer questions on a general classroom level like “if a teacher thinks s/he is generally more supportive (compared to other teachers), does s/he have students who feel more socially included, who have more egalitarian hierarchy and generally more academically engaged students?”

Based on scores for each outcome variable, individual student scores will be predicted using the relationship variance components (e.g., teacher relationship and student relationship effects; see Table 3). This way we can answer questions on an individual dyadic level like “if a student thinks s/he is generally more emotionally supported by her/his teacher (compared to other students), does s/he feel more socially included and feel generally more academically engaged?”

### Differences by country

In all of the above analyses country will be added as a covariate in order to check for differences between countries.

## Prospective discussion

The proposed study will use a dyadic analysis called the one-with-many design in examining teacher–student relationships, which no other previous study has done before. This way of assessing teacher–student relationships will provide a wealth of information which have not yet been examined: By looking at the reciprocity of student and teacher reports of teacher emotional support, we can assess the importance of student's perceived emotional support on academic functioning and social inclusion. Therefore, this study will extend the literature on teacher–student relationships by including measures from the other, and equally important, part of the relationship, being the student.

Previous studies have shown that teacher support promotes academic competence and prevents problematic behaviors in the classroom (Tennant et al., 2014). However, the importance of teacher support for children's social well-being remains unknown (Farmer et al., 2011). With the dyadic analysis, perceptions of consensus between student and teacher reports could explain why certain children feel more socially included and why certain teachers establish especially inclusive social climates in their classrooms.

Accordingly it is anticipated that high teacher perceiver and partner variances—i.e., high generalized reciprocity—will predict social inclusion and classroom climate. In classrooms with low teacher perceiver and partner variances we expect to still see high relationship variances—i.e., high dyadic reciprocity—which would indicate specific dyads with uniquely strong emotional support and hence better student outcomes for the specific students involved in these dyads. Regarding Marcus et al. (2011) study in which high relationship variances were found for the therapists and substance use adolescents in regard to therapeutic alliance (Marcus et al., 2011), we do not expect such effects in the present study. As therapeutic alliances are dyadic by nature, classroom interactions between a teacher and multiple students are less likely to be denoted by such relationships as a teacher mostly interacts with the whole classroom at any given time. Hence generally we would expect higher teacher perceiver and partner variances and generalized reciprocity as opposed to relationship variances and dyadic reciprocity to reveal the general nature of classroom interactions. Due to the general similarity of the two countries where data collection has been conducted we do not expect any country differences.

Limitations exist for the one-with-many design. Since each student has one single teacher, it is not possible to completely isolate teacher partner effects. That means that it is not entirely clear whether students would report similar emotional support had they been educated by more than one teacher. Similarly it is not entirely clear whether different teachers would report similar emotional support had they all been educating the same student. Still the partial variance partitioning provided by an one-with-many design is superior to analyses that ignore the nestedness of students Future research that includes the perceptions of more than one teacher per classroom (potentially teachers of other subjects that also teach the same class) could make an even better use of such a design.

Assuming the current study finds effects of teacher's emotional support on children's social inclusion dependent on teacher–student relationships, it will highlight a new area of intervention. For instance, policies to improve teacher training or school interventions can be discussed to achieve inclusive classroom climates, which further can lead to better academic performance and increased well-being.

Sample size restrictions should be viewed in light of several practical limitations concerning recruitment, data collection and questionnaire administration. In a similar fashion, theoretical concerns regarding study design and power estimates need to be considered.

To begin with, recruitment and data collection were subject to ethical approval procedures. The imposed requirement to obtain active consent instead of passive parental consent resulted in delays in receiving ethical approval from the main investigator's University. Following this, a similar process of obtaining approval was undertaken and consequently delayed in each country participating in the study. In regards to parental consent, sample size was affected by factors such as refusal to participate or inability to return the completed forms. In some cases, children decided not to take part anymore or refused to complete the whole questionnaire. Consequently, these pitfalls affected the sample size at the lower level in particular.

Secondly, limitations regarding questionnaire administration have so far been identified and should be discussed. All participating pupils were asked to complete a 15-page long questionnaire. As previously mentioned, the tool had been previously piloted in English, Romanian as well as Turkish and the estimated time for completion ranged between 30 and 45 min for each language. In practice, the time allocated for data collection per classroom was set for approximately an hour. The time varied from one classroom to another, however it never exceeded the allocated time slot. Specific issues were identified with the sociometric questions. The coding system, which was set in place in order to anonymise the answers and facilitate peer nominations analysis, was difficult to understand for some participants. This is likely to have caused fatigue and boredom. Nonetheless, the questionnaire was designed in a way that would counter for the aforementioned boredom effects. In this sense, a storyline that was appealing to students was introduced prior to questionnaire completion and it was maintained throughout the whole process. Students were all told they were on a secret mission to help a team of minions find valuable information about their classroom. The response to the story was always positive and ensured students' commitment to and focus on the task. With the exception of peer nominations and the teacher–student relationship items, all questions employed a smiley-face rating scale which easy to understand and use. Moreover, each questionnaire was fairly short in length with items varying in number from 10 (academic engagement; Skinner et al., 2008) to 26 (SIAI; Rinta et al., 2011). Finally, jokes and pictures of minions (famous cartoon characters) were included to break down the length of the questionnaire or the potential monotony.

The employed design is classified as cross-sectional as the study is conducted at one time point only. No causality could thus be inferred, however this type of design serves the aim of our research with regards to exploring the predictive nature of perceived emotional support on students' social inclusion. In addition to this, the study is designed to be multimodal and multi-informant. This is advantageous as multi-informant data is valuable in terms of obtaining a more accurate description of the studied phenomena. In practice, by gathering data about peer relationships, teacher–student relationships, classroom climate, social inclusion and academic engagement and performance, we are able to draw on a detailed depiction of classroom dynamics and their outcomes.

Finally, limitations also exist in the estimation of sample size. Generally the requirement with hierarchical data as opposed to leveled data is that the more levels there are the more parameters need to be estimated which make a priori estimations challenging as controversial discussions on the sample size estimation in MLM show (Field, 2013). Nevertheless the above standard power analyses has been run to demonstrate that sufficient power can be provided with our sample size aim. The neglect of multiple levels and slight underpower of 0.7 constitute a limitation of this simplified estimate. Though this calculation as well as any limitations of it need to be qualified by the complexity of sample size estimations in multilevel models in general, which caused many in practice to arrive to the rule of the more data, the better (Kreft and Leeuw, 1998; Field, 2013) as statistical power analyses are not traditionally being carried out for multilevel models. Another general rule with multilevel models that are reported in literature append more emphasis to the group level sample size as opposed to the sample size of individuals within groups (Snijders, 2005; Twisk, 2006), i.e., the number on the group level is more important. More concrete estimates are provided by simulation studies stating that sample size greater than 30 have little impact on the accuracy of standard errors of fixed effects and advocate said number as normal in educational research to achieve sufficient power (Maas and Hox, 2005).

To conclude, this research will contribute to the use of psychological assessment in educational settings by introducing new methods for measuring emotional support, social inclusion and academic engagement from the view of the student and the teacher.

## Author contributions

YV supervised the project, the paper and supported team members with expertise. ZH wrote the abstract, parts of the introduction, proposed analyses, and discussion. LH wrote the methods and proposed analyses. JB was in charge of the introduction and abstract. MÇ contributed to the introduction as well as prospective discussion. MCC recruitment in the UK and co-wrote abstract, introduction, methods and discussion AB was in charge of recruitment in Turkey and ethics.

## Funding

This research was supported in part by the University of Oslo, Radboud University, and University of Worcester. The authors gratefully acknowledge the financial support which facilitated our data collection and dissemination plans.

### Conflict of interest statement

The authors declare that the research was conducted in the absence of any commercial or financial relationships that could be construed as a potential conflict of interest.

## Appendix

### SPSS syntax to estimate the variance partitioning

**MIXED**

TSR WITH TEACHER STUDENT

**/FIXED** = TEACHER STUDENT|**NOINT**

**PRINT** = **SOLUTION TESTCOV**

**RANDOM** TEACHER STUDENT|**SUBJECT**(TEACHERID)

**                                                                 COVTYPE(UNR)**

**REPEATED** = ROLE|**SUBJECT**(TEACHERID ^*^ STUDENTID)

**                                                                   COVTYPE(UNR)**

Unbolded terms represent variable names. The two dummy codes for teacher (TEACHER) and student (STUDENT) are needed to estimate a two intercept model as stated in the proposed analysis above. Hence two separate intercepts are estimated for teacher and students by simultaneously suppressing the traditional intercept (i.e., NOINT).

The RANDOM statement results in estimation of the variance in the two intercepts. For teachers this variance estimates the teacher perceiver variance. For the students this variance estimates the teacher partner variance. Covariance is estimated by the COVTYPE term. UNR specification of that term means that instead a correlation between teacher perceiver and teacher partner is obtained.

The REPEATED statement is needed to specify the relationship variances. COVTYPE(UNR) obtains the correlations of between these variances.
